# A new highly cave-adapted trechine genus and species from northern Guizhou Province, China (Coleoptera, Carabidae, Trechinae)

**DOI:** 10.3897/zookeys.643.11050

**Published:** 2017-01-06

**Authors:** Mingyi Tian

**Affiliations:** 1 Department of Entomology, College of Agriculture, South China Agricultural University, No. 483, Wushan Road, Guangzhou, 510642, China

**Keywords:** aphaenopsian, ground beetle, hypogean, southern China Karsts

## Abstract

A remarkable aphaenopsian beetle, a sympatric species of *Qianotrechus tenuicollis* Uéno, 2000, was newly discovered in Cave Mahuang Dong of Shuanghe Dong cave system, the longest cave system of China in Suiyang County, northern Guizhou Province. To categorize this striking but still unknown species, a new genus and species are proposed: *Shuangheaphaenops elegans* **gen. n.**, **sp. n.** Relationships of *Shuangheaphaenops* and other highly modified aphaenopsian genera from southern China Karsts are discussed.

## Introduction

Guizhou Province and Guangxi Zhuang Autonomous Region of southern China hold the largest karstic landscapes in the world (Waltham 2009), and the richest cave beetle fauna, too, at least at generic level. Of 120 hypogean trechine species belonging to 48 genera known in China (Deuve and Tian 2016; Fang et al. 2016; Tian et al. 2016; Zhao and Tian 2016), 72 species in 23 genera are reported from Guizhou and Guangxi karsts. Furthermore, all highly modified aphaenopsian genera are distributed only in southern Guizhou and northern Guangxi, such as *Sinaphaenops* Uéno & Wang, 1991, *Dongodytes* Deuve, 1993, *Giraffaphaenops* Deuve, 1992, *Pilosaphaenops* Deuve & Tian, 2008 and *Uenotrechus* Deuve & Tian, 1999 (Deuve et al. 1999).

Members of *Sinaphaenops* are endemic to southern parts of Guizhou, with only an exception for *Sinaphaenops wangorum* Uéno & Ran, 1998 which is recorded from both Libo (southernmost Guizhou) and Huanjiang (northernmost Guangxi) counties (Uéno and Wang 1991; Uéno and Ran 1993; Uéno 2002; Tian et al. 2010; Deuve and Tian 2014; Tian and Huang 2015). All species of both *Pilosaphaenops* and *Uenotrechus*, together with three species of *Sinaphaenops*, are occurring in a narrow area between southernmost Guizhou and northernmost Guangxi, viz. Maolan, Mulun and adjacent karstic areas, where the largest and most primitive karstic forest in southern China is maintained (Deuve et al. 1999; Deuve and Tian 2008; Uéno 2009; Tian 2010, 2011). The genera *Dongodytes* and *Giraffaphaenops* are endemic to northern Guangxi, but not sympatric with above three genera (Deuve 1993, 2002; Uéno 1998, 2005; Tian and Luo 2015).

In October, 2016, a cave biodiversity survey was carried out by our team in several counties of Zunyi and Tongren Districts, northern and northeastern Guizhou, in order to collect species of the genera *Qianaphaenops* Uéno, 2000 and *Qianotrechus* Uéno, 2000. The result was very satisfactory, leading to increase the number of almost all of the Uéno’s species recorded from these areas. In addition, an unexpected single male beetle and an elytral debris of the same species were collected in a limestone cave belonging to Shuanghe Dong cave system, Suiyang County. This extremely troglobiomorphic aphaenopsian beetle looks like a *Uenotrechus* species at first sight on its fore body (head and thorax), but the hind part (elytra) is more likely similar to a *Dongodytes* (*s. str.*) species. It has three pairs of supraorbital setiferous setae on head and lacks lateromarginal setae on pronotum. It is also different from *Sinaphaenops* Uéno & Wang although its first and second male protarsomeres are dilated and spurred inward apically, a sexual modification appeared also in some *Sinaphaenops* species. Far more important, its elytral chaetotaxy is not similar to any other aphaenopsian genera mentioned above. Therefore, this interesting beetle represents a peculiar lineage within Chinese hypogean trechines. Here we describe this remarkable species, the first highly cave-adapted aphaenopsian species from northern Guizhou Province.

As the longest cave system in China, Shuanghe Dong is connected by 42 caves or entrances, with total length coming up to 186.33 km (He et al. 2016). But the major part of this cave system is still not investigated regarding cave biodiversity. Hence, it is expected that more hypogean trechine beetles would be discovered from this cave system in future.

## Materials and methods

The single blind beetle and the elytral debris for this study were collected by the naked eye using an aspirator inside the cave Mahuang Dong, and kept in 50% ethanol before study. Other cave beetles used for comparing were dry and mounted specimens of the insect collection of South China Agricultural University, Guangzhou, China (SCAU).

Dissections and observations were made under a Leica S8AP0 microscope. Dissected genital pieces, including the median lobe and parameres of the aedeagus, were glued onto small transparent plastic plates and pinned under the specimen. Habitus pictures were taken by means of a Keyence VHX-5000 digital microscope. Genital pictures were taken using a Canon EOS 40D camera connected to a Zeiss AX10 microscope, and then stacked and processed using Adobe Photoshop CS5 software. Distribution maps were drawn using Mapinfo software.

The length of the body was measured from the apex of the right mandible (in open position) or from labrum to the elytral apex; the width of the body was taken as the maximum width of the elytra.

Abbreviations of other measurements used in the text are as follows:

HLm
 length of head including mandibles, from apex of right mandible to neck constriction 


HLl
 length of head excluding mandibles, from front of labrum to occipital suture 


HW
 maximum width of head 


PrL
 length of prothorax, along the median line 


PnL
 length of pronotum, as long as PrL 


PrW
 maximum width of prothorax 


PnW
 maximum width of pronotum 


PfW
 width of pronotum at front 


PbW
 width of pronotum at base 


EL
 length of elytra, from base of scutellum to elytral apex 


EW
 maximum width of combined elytra 


## Taxonomic treatment

### Genus Shuangheaphaenops gen. n.

Taxon classificationAnimaliaColeopteraCarabidae

http://zoobank.org/0E7CBC8A-C92E-426F-A331-93156EE2B0A8

#### Type species.

*Shuangheaphaenops elegans* sp. n.

#### Diagnosis.

Large sized blind beetles, fore body evidently elongated and as long as elytra, shape intermediate between *Uenotrechus* and *Dongodytes* species, presence of three pairs of supraorbital setae on head, two dorsal and preapical pores on elytra, the first and second protarsomeres in male distinctly modified.

#### Generic characteristics.

Highly modified aphaenopsian trechines, fore part (head and thorax) of the body somewhat similar to *Uenotrechus* Deuve & Tian, 1999, while hind part (elytra) to *Dongodytes* Deuve, 1993; large sized, with body and appendages thin and very elongate, fore body almost as long as hind part; three pairs of supraorbital setiferous pores present on head, with the posterior two pairs very close to each other; mandibles thin and elongated, feebly curved apically, longer than width of head, right mandible edentate though two vanished teeth can be faintly traced; labial suture moderately defined, separating of mentum and submentum, with the former bisetose and the latter 6-setose; mental tooth simple and thin, basal foveae quite narrow; antennae very long, the 10^th^ and 11^th^ antennomeres extending over apical margin of elytra. Prothorax dolioform, propleura distinctly tumid at basal half, evidently visible from above; pronotum barrel-shaped, distinctly elongated, longer than head excluding mandibles, narrower than head; without lateromarginal setae. Elytra similar to those of *Dongodytes* (*s. str.*) Deuve, 1993, narrowed anteriorly and dilated posteriorly, side margins narrowly bordered throughout, shoulders lacking; striae lacking though easily traceable; presence of two dorsal and preapical setiferous pores; the 1^st^ pore in the humeral group of the marginal umbilicate series not transversely and backwardly shifted, the 5^th^ and 6^th^ pores in the middle group widely spaced. Protibia smooth, without longitudinal sulcus; the 1^st^ and 2^nd^ protarsomeres in male dilated and inwardly spurred at apices. Abdominal ventrites sparsely pubescent, each of ventrites IV-VII in male bisetose apically. Male genitalia moderately sclerotized, small, strongly curved ventrally in lateral view, with a quite large sagittal aileron; apical lobe very thin in dorsal view; parameres well developed, but much shorter than median lobe.

#### Discussion.

*Shuangheaphaenops* can not be included in any lineage of the highly modified aphaenopsian genera known in southern China regarding to the peculiar morphological characteristics mentioned above, such as the peculiar facies and configuration of the body (which is more or less similar to *Uenotrechus* Deuve & Tian, 1999 in fore body, but to *Dongodytes* Deuve, 1993 in elytra), vanished bidentate right mandible, and chaetotaxal patterns in which there are three pairs of supraorbital setiferous pores on head, lack of lateromarginal setae on pronotum, and unique pattern on elytral marginal umbilicate series, in particular, the humeral and middle groups.

Apart from the similarity in elytra and antennae between *Shuangheaphaenops* and *Dongodytes* (*s. str.*) which occurs only in northern Guangxi where is far distant from Cave Shuanghe Dong in northern Guizhou, the following characteristics are different: (1) head subparallel-sided, with three pairs of supraorbital setiferous pores, right mandibular teeth bidentate but almost vanished in *Shuangheaphaenops* (versus triangular shaped in general, presence of two pairs of supraorbital pores, and well-marked tridentate teeth on right mandible in *Dongodytes*); (2) the 1^st^ and 2^nd^ protarsomeres of male distinctly modified in *Shuangheaphaenops* (indistinctly or not modified in *Dongodytes*); (3) pronotum much slender and lack of lateromarginal setae in *Shuangheaphaenops* (versus stouter and presence of lateromarginal setae in *Dongodytes*); and (4) the middle group (the 5^th^ and 6^th^ pores) of the marginal unbilicate series on elytra widely spaced each other in *Shuangheaphaenops* (versus close to each other in *Dongodytes*).

The fore body of this new genus is more or less similar to that of *Uenotrechus* Deuve & Tian, 1999, but *Shuangheaphaenops* has a slenderer head bearing three pairs of supraorbital setiferous pores, reduced bidentate teeth of right mandible, and much longer antennae (versus bearing two pairs of supraorbital setiferous pores, mandibular teeth well-defined and clearly tridentate, and shorter antennae in *Uenotrechus*), and pronotum without lateromarginal setae (versus with pair of lateromarginal setae in *Uenotrechus*). In addition, head and elytra are glabrous in *Shuangheaphaenops* (versus whole body densely pubescent in *Uenotrechus*), the 1^st^ pore of the marginal umbilicate series is located before the 2^nd^ in *Shuangheaphaenops* (versus transversely shifted inwards and backwards, at level behind the 2^nd^ pore in *Uenotrechus*), and both the 1^st^ and 2^nd^ protarsomeres in male are modified in *Shuangheaphaenops* (versus not modified in *Uenotrechus*).

#### Etymology.

“Shuanghe + Aphaenops”. To indicate that the highly modified trechine genus occurs in Shuanghe Dong, the longest cave system in China.

#### Generic range.

**Figure 1.**
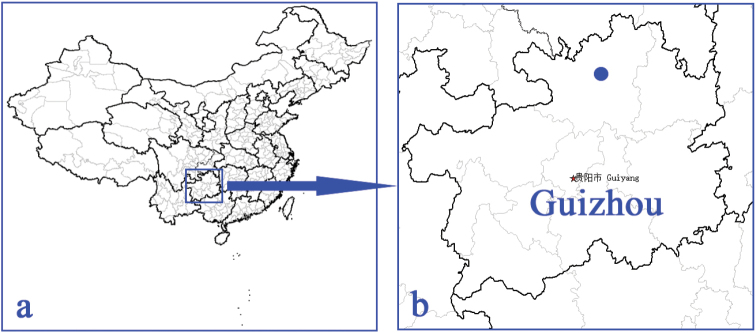
Distribution of *Shuangheaphaenops elegans* gen. n., sp. n. **a** map of China showing the location of Guizhou Province **b** map of Guizhou Province, the location of Shuanghe Dong cave system shown by blue point.

Guizhou (Suiyang) (Fig. 1).

### Shuangheaphaenops elegans sp. n.

Taxon classificationAnimaliaColeopteraCarabidae

http://zoobank.org/392C6535-5261-47FF-A2F7-D7123A5D9DFA

**Figure 2.**
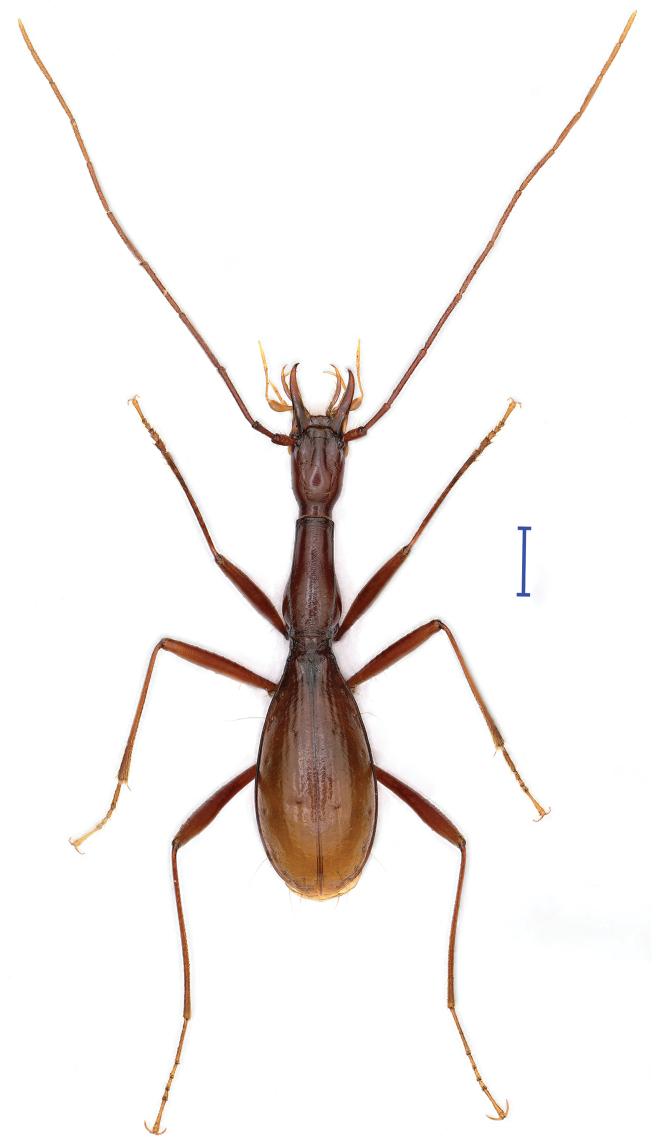
Habitus of *Shuangheaphaenops elegans* gen. n., sp. n., holotype, male. Scale bar 1 mm.

**Figure 3.**
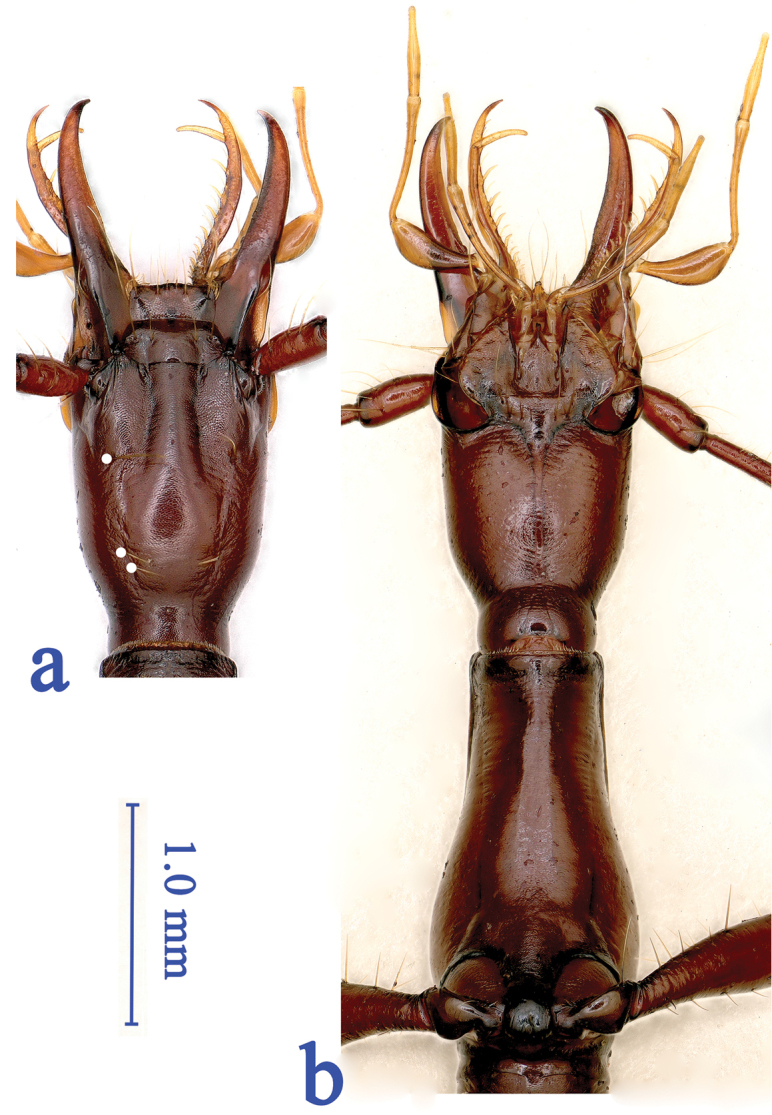
Head and prothorax of *Shuangheaphaenops elegans* gen. n., sp. n. **a** head, dorsal view, supraorbital setiferous pores on the left side shown by white points **b** head and prothorax, ventral view.

**Figure 4.**
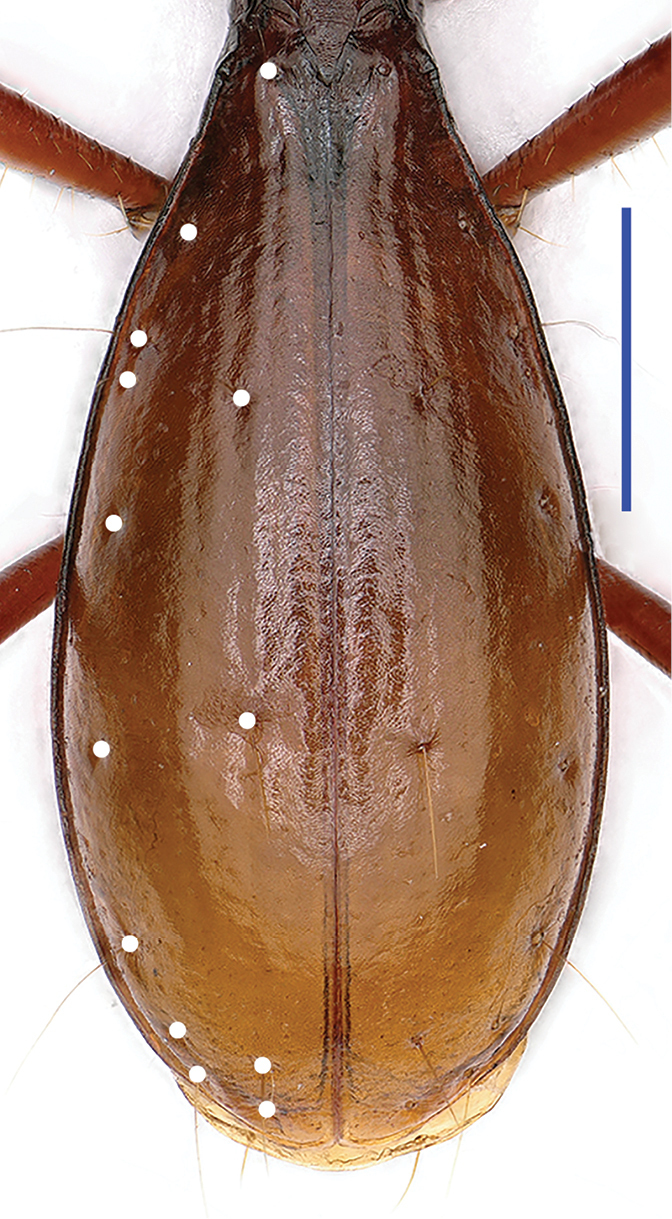
Elytra of *Shuangheaphaenops elegans* gen. n., sp. n., holotype, male, chaetotaxal pattern shown by white points. Scale bar 1 mm.

**Figure 5.**
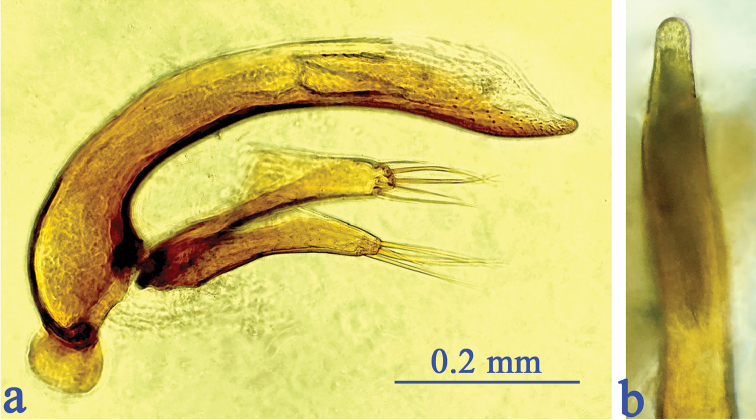
Male genitalia of *Shuangheaphaenops elegans* gen. n., sp. n. **a** median lobe and parameres, lateral view **b** apical lobe, dorsal view.

**Figure 6.**
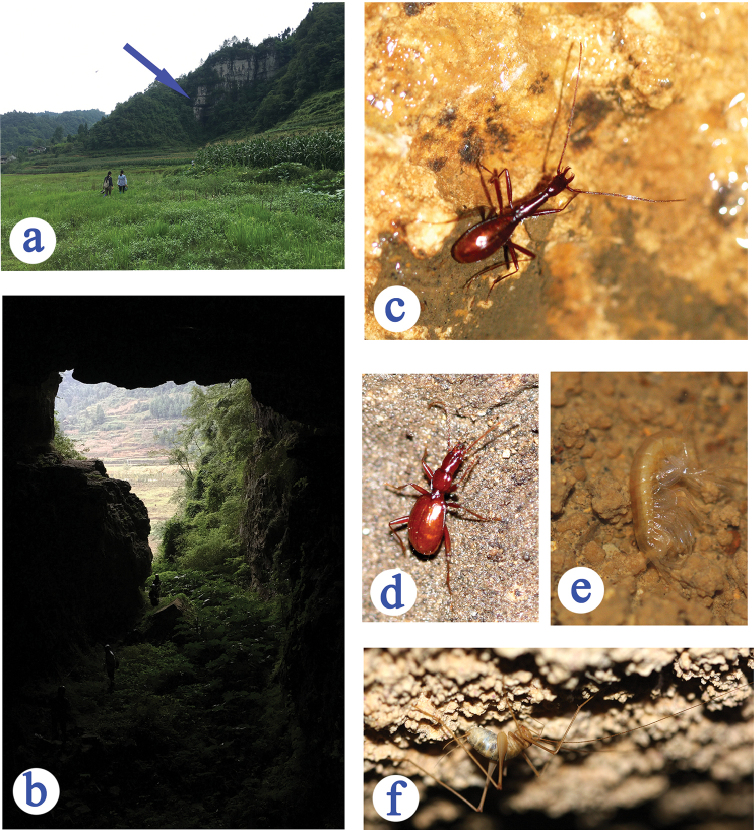
Cave Mahuang Dong, the type locality of *Shuangheaphaenops elegans* gen. n., sp. n., and some sympatric cave animals **a** opening, showed by arrowhead **b** entrance **c** *Shuangheaphaenops elegans* **d** a *Qianotrechus tenuicollis* Uéno, 1998 **e** amphipod **f** cave cricket.

Figs 1 , 2 , 3 , 4 , 5 , 6

#### Holotype.

Male, Cave Mahuang Dong, Shuanghe Dong cave system, Wenquan Zhen, Suiyang County, 28°14'32"N, 107°17'24"E, 720 m, X-18-2016, leg. Wenbo Li, deposited in the insect collection of South China Agricultural University, Guangzhou, China (SCAU); additional material: an elytral debris, same cave and collecting date as above, leg. Mingruo Tang, in SCAU.

#### Diagnosis.

A large-sized, eyeless cave trechine beetle, highly modified in morphology, with very elongated and slender body which is about four times longer than wide, fore body about as long as elytra, antennae as long as body including mandibles, extending beyond elytral apex; body glabrous, except for basal half of pronotum which is covered with erected setae.

#### Description.

Length: 7.9 mm (from apex of right mandibles to elytral apex) or 7.2 mm (from labrum to elytra); width: 1.79 mm. Fore body (including mandibles) longer than elytra, (HLm+PrL)/EL = 1.07. Habitus as in Fig. 2.

Yellowish brown, a little darker on head, pronotum and basal half of elytra, pale on antennae, mouthparts, palps and tarsi. Moderately shiny. Head and elytra glabrous, pronotum glabrous on apical half (but with two or three short setae near middle of frontal margin) but pubescent on basal half. Underside of head and prothorax glabrous (Fig. 3b), abdominal ventrites sparsely pubescent. Microsculptural engraved meshes more or less isodiametric on head and elytra, but transverse striate on pronotum.

Head (Fig. 3a) elongate quadrate, much longer than wide (HLm/HW = 2.74, HLl/HW = 1.87); genae fairly developed, hardly dilated laterally, suddenly constricted posteriorly before occipital suture, making a well-marked but short neck constriction; subparallel-sided, widest at about middle from labrum to base of head; frons and vertex moderately convex, frontal furrows deep and well-marked, subparallel-sided, ended just behind the level of anterior supraorbital pores; clypeus transverse, 4-setose; labrum transverse, with frontal margin slightly protruding medially, 6-setose; anterior supraorbital setiferous pores located at about middle from frontal margin of labrum to base of head, while the posterior two pairs (which are very close to each other) at about 1/4 of head from base; palps long, slender and glabrous except for the 2^nd^ labial palpomere which is bisetose on inner margin; the 2^nd^ labial palpomere 1.35 times longer than the 3^rd^; the 3^rd^ maxillary palpomere 1.25 times longer than the 4^th^; suborbital pores at about middle from base to labial suture. Antennae thin and long, the 1^st^ antennomere stouter than other, and the shortest, the 3^rd^ the longest; the comparative length ratio of each antennomeres as: the 1^st^ (8.5), 2^nd^ (11.0), 3^rd^ (18.0), 4^th^ (17.0), 5^th^ (17.0), 6^th^ (16.5), 7^th^ (13.5), 8^th^ (11.0), 9^th^ (11.0), 10^th^ (10.0) and 11^th^ (10.5).

Prothorax shorter than head including mandibles (PrL/HLm = 0.80), but longer than head excluding mandibles (PrL/HLl = 1.17), widest at about 1/4 from base, twice as long as wide (PrL/PrW = 2.10), slightly wider than head (PrW/HW = 1.09), evidently wider than pronotum (PrW/PnW = 1.22), half as wide as elytra (PrW/EW = 0.49). Pronotum elongate, dolioform, two and half times longer than wide (PnL/PnW = 2.46), evidently narrower than head (PnW/HW = 0.89), base slightly wider than front (PbW/PfW = 1.09); lateral sides finely bordered throughout, base and front unbordered; nearly parallel-sided, fairly expanded at the widest part which is at about 3/7 from base, slightly sinuate before hind angles which are nearly rectangular, fore angle obtuse; median line well-marked, basal transversal impression very short; front slightly convex, base feebly concave. Scutellum fairly small.

Elytra (Fig. 4) elongate ovate, much longer than pronotum (EL/PnL = 2.12), about twice as long as wide (EL/EW = 2.08); distinctly dilated posteriorly, widest at about apical 3/7 of elytra, lateral sides smooth, not ciliate, finely bordered throughout; distinctly convex; striae easily traceable though devoid, intervals moderately convex. Chaetotaxy: basal pore at subequal distant from scutellum to marginal gutter; two dorsal setiferous pores present on the 3^rd^ stria at about 1/3 and 2/3 from base respectively; the preapical pore closer to suture than to apical margin; the marginal umbilicate pores not aggregated, the 8^th^ pore near marginal gutter, the 4^th^ and 5^th^ pores distant from the gutter, others intermediary located; the humeral groups separately spaced, the 1^st^ and 4^th^ pores distant from the 2^nd^ and 3^rd^ pores respectively which are close to each other, distance from the 1^st^ pore to the 3^rd^ slightly shorter than that from the 2^nd^ to 4^th^; the middle group widely spaced, making distance of the 5^th^ pore and 4^th^ subequal to that of the 5^th^ and 6^th^; apical group composed of three pores.

Legs thin and long, bearing short pubescence; fore- and middle femora sparsely setose; fore tibia smooth, without longitudinal furrow or sulcus; the 1^st^ tarsomere shorter than, as long as, and longer than the 2^nd^–4^th^ tarsomeres together in fore, middle, and hind legs, respectively.

Male genitalia (Fig. 5): Aedeagus moderately sclerotized, quite small and short; distinctly curved at about basal 1/3 in lateral view, feebly curved toward subapex, then broadly ended at apex; inner sac armed with a fairly large copulatory piece which is about 1/4 as long as the median lobe; base moderately sized, opened ventrally; in dorsal view the apical part of aedeagus thin, slightly sinuate from middle to apex, apical lobe narrow, much longer than wide, gradually constricted towards the rounded apex. Parameres short and quite stout, right and left parameres bearing five and four long apical setae respectively.

#### Etymology.

To indicate the slender shape of this beautiful aphaenopsian beetle.

#### Distribution.

China (Guizhou: Suiyang) (Fig. 1). Known only from Cave Mahuang Dong, the type locality.

Mahuang Dong (Figs 6a, b) is one of the most important caves in Shuanghe Dong cave system (Li et al. 2008). It is opened along the main road of the Shuanghe Dong National Geopark on north, and is about 1.2 km long. The beautiful beetle, together with the elytral debris, were collected in a small chamber of the labyrinthic part at about 100 m from the entrance. Other cave animals found also in Mahuang Dong were the semi-aphaenopsian beetle *Qianotrechus tenuicollis* Ueno, 1998, an amphipod, a cave cricket (Figs 6c-f), a pseudoscorpion and two millipedes.

## Supplementary Material

XML Treatment for Shuangheaphaenops

XML Treatment for Shuangheaphaenops elegans
